# Cell Cycle and Anti-Estrogen Effects Synergize to Regulate Cell Proliferation and ER Target Gene Expression

**DOI:** 10.1371/journal.pone.0011011

**Published:** 2010-06-08

**Authors:** Mathieu Dalvai, Kerstin Bystricky

**Affiliations:** 1 Université de Toulouse, UPS, Laboratoire de Biologie Moléculaire Eucaryote, Toulouse, France; 2 CNRS, Laboratoire de Biologie Moléculaire Eucaryote, Toulouse, France; Garvan Institute of Medical Research, Australia

## Abstract

Antiestrogens are designed to antagonize hormone induced proliferation and ERα target gene expression in mammary tumor cells. Commonly used drugs such as OH-Tamoxifen and ICI 182780 (Fulvestrant) block cell cycle progression in G0/G1. Inversely, the effect of cell cycle stage on ER regulated gene expression has not been tested directly. We show that in ERα-positive breast cancer cells (MCF-7) the estrogen receptor gene and downstream target genes are cell cycle regulated with expression levels varying as much as three-fold between phases of the cell cycle. Steroid free culture conditions commonly used to assess the effect of hormones or antiestrogens on gene expression also block MCF-7 cells in G1-phase when several ERα target genes are overexpressed. Thus, cell cycle effects have to be taken into account when analyzing the impact of hormonal treatments on gene transcription. We found that antiestrogens repress transcription of several ERα target genes specifically in S phase. This observation corroborates the more rapid and strong impact of antiestrogen treatments on cell proliferation in thymidine, hydroxyurea or aphidicolin arrested cells and correlates with an increase of apoptosis compared to similar treatments in lovastatin or nocodazol treated cells. Hence, cell cycle effects synergize with the action of antiestrogens. An interesting therapeutic perspective could be to enhance the action of anti-estrogens by associating hormone-therapy with specific cell cycle drugs.

## Introduction

Estrogens play a key role in the development of the mammary gland. In the normal gland, proliferating cells do not express estrogen receptors. In contrast, estrogen receptor-α (ERα) which acts as a ligand (estrogen)-dependent transcription factor is expressed in the majority of mammary tumors (70%). Recent transcriptome analyses confirmed observations made over a century ago, that estrogens stimulate the development of the disease in at least one out of five patients [1], [2], [3].

The control of cell proliferation by estrogens such as 17-ß estradiol (E2) is a complex process. Estrogens bound to ERα regulate target genes implicated in proliferation including *CDK2*, *CDK4*, Cyclin D1 (*CCND1*) or the proto-oncogene c-Myc (*MYC*) [4], [5], [6]. In addition, several genes which negatively control cell proliferation such as cyclin G2 (*CCG2*), caspase 9 (*CSP9*) or *p21* are repressed by estrogens [7], [8]. Rapidly and transiently, estrogens activate signal transduction pathways, acting in particular through mitogen-activated protein kinases (MAPK) [9], [10]. The fact that estrogens promote tumorigenesis has led to the development of anti-hormone therapies. Synthetic compounds that either act as estrogen-antagonists or block the function of aromatases (the enzymes that catalyze the last step of estrogen biosynthesis) have been designed. Several publications also reported an effect of anti-estrogens on expression and/or intracellular distribution of factors that regulate cell cycle progression. Anti-estrogens such as Tamoxifen (OH-TAM) or ICI 182.780 (ICI) block ERα-positive breast cancer cells in G1 [11], [12].

The effects of estradiol and hormone-therapy on cell cyle progression are very well documented [12], [13], [14], [15] showing that variations in ERα target gene expression largely influence cell cycle regulators, including cyclins. In contrast little is published on the variation of *ESR1* and ERα target gene expression during the course of the cell cycle. Previously, only expression of the progesterone receptor gene (*PGR*) was studied during the cell cycle in T47D cells [16].

In this study, we analysed commonly studied ERα target genes involved in cell differentiation, such as *TFF1* (pS2), estrogen receptor (*ESR1*) or *PGR*, and in cell proliferation, such as Cathepsine-D (*CTSD*) and *CCND1*. We also included the gene coding for the histone variant H2A.Z in our analysis. This gene is indirectly regulated by ERα via *c-myc*, which leads to an increase in transcription and H2A.Z protein synthesis in MCF-7 breast cancer cell lines [6], [17]. We have investigated whether the effect of several commonly used antiestrogens such as OH-TAM and ICI on ERα target gene expression and cell proliferation was dependent on the cell cycle stage. We demonstrate that transcription of all ERα target genes analysed is cell cycle regulated and that antiestrogens and culture conditions affect cell cycle progression. We further show that cell cycle effects influence the action of antiestrogens. In particular, we found that the effect of OH-TAM and ICI was significantly enhanced in cells blocked in S phase by reduction of gene expression and an increase in apoptosis.

## Results

### Estrogen-regulated gene expression is cell cycle regulated

**Figure 1:**
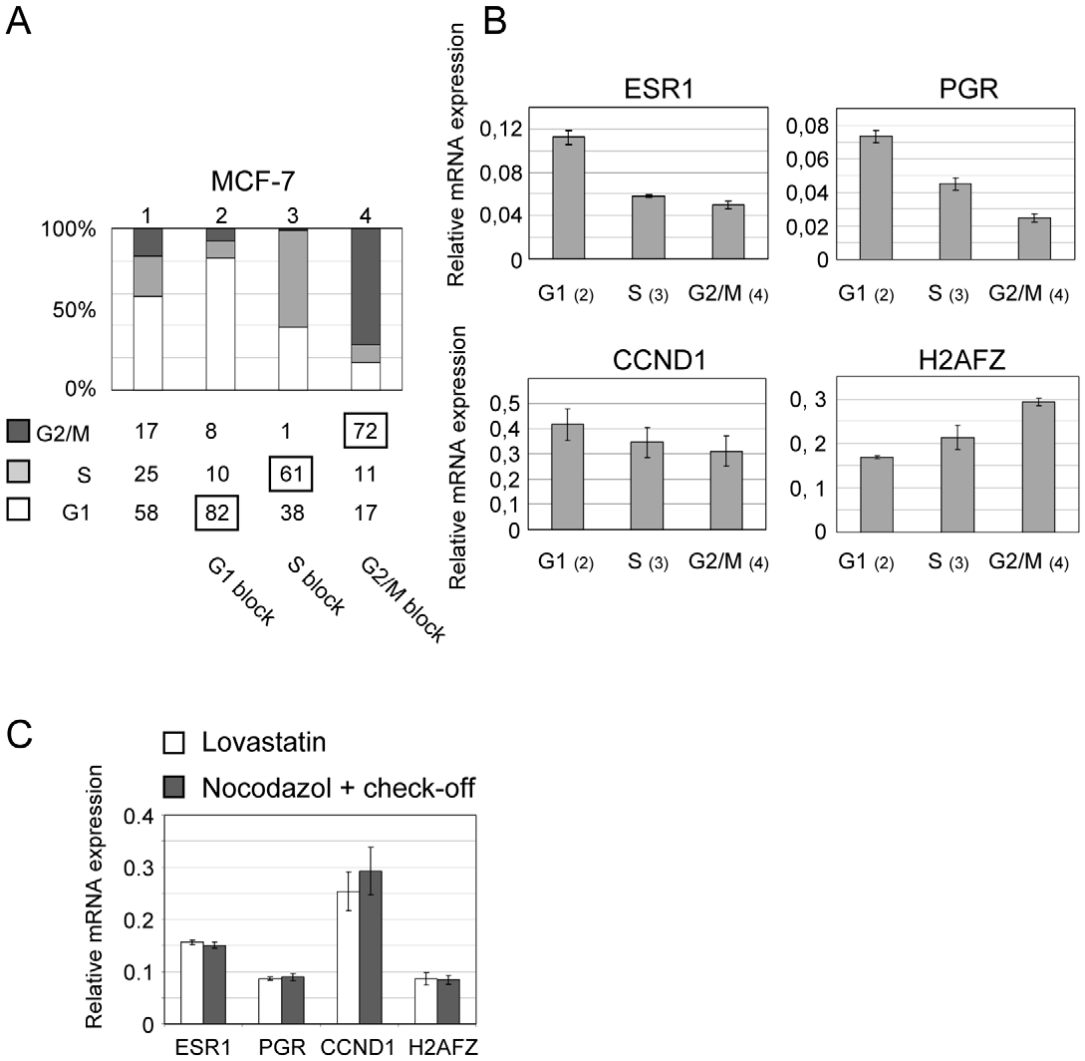
ERα target gene expression is cell cycle regulated in MCF-7 cells. A) FACS analysis after propidium iodide staining on asynchronous untreated cells (1), cells blocked in G1 by lovastatin treatment 20 µM for 32 h (2), in S phase by thymidine treatment 3 mM for 24 h (3) or in G2/M by nocodazol 50 ng/ml for 24 h (4). B) Real-time PCR analysis of *ESR1*, *PGR*, *CCND1* and *H2AFZ* gene expression in G1 (lovastatin), S (thymidine) and G2/M (nocodazol) cell cycle phases. 2×10^6^ of MCF-7 cells were seeded in 10 cm dishes and after 24 h submitted to specific cell cycle arrest drugs as in (A). Total RNA was extracted and reverse transcribed. The amount of analyzed genes cDNA was measured RT-qPCR divided by the amount of RLP0 cDNA. (n = 3) one representative experiment is shown. C) Real-time PCR analysis of *ESR1, PGR, CCND1* and *H2AFZ* gene expression. For lovastatin treatment cells were treated as in (B). For nocodazol/check-off treatment, 12×10^6^ cells were splited into two 140 cm dishes. After 24 h of nocodazol treatment (25 ng/ml), G2/M arrested cells are harvested by check-off.and seeded in a clean dish. After 7 hours in complete medium total RNA was extracted and reverse transcribed (n = 2).

Under standard growth conditions, 58% of non confluent MCF-7 cells are in G1, 25% in S and 17% in G2 phase (Figure 1A, column 1) in the absence of cell cycle modifying drugs. MCF-7 cells were exposed to 20 µM lovastatin for 32 hours, 3 mM thymidine or 50 ng/ml nocodazol for 24 hours. FACS analysis confirmed that lovastatin (column 2) synchronized cells in G1 (82%), that a thymidine treatment (column 3) induced an S-phase block (61%) and that nocodazol (column 4) lead to an arrest in G2/M (72%) compared to asynchronous cells (column 1). We chose lovastatin and thymidine over other cytotoxic compounds for their limited toxicity. Indeed, minimal perturbations of general metabolic functions occurred since the rate of RNA, protein, and initial DNA synthesis were unaffected by Lovastatin [18]. In particular, the use of 10 nM ICI, frequently found in the literature to synchronize cells in G1 could not be employed for our study since ICI is not neutral when examining ERα regulated gene expression. We thus chose to synchronize cells in S-phase using thymidine since this method is one of the most effective and most widely used techniques. The rationale of this method based on high concentrations of thymidine inhibiting ribonucleotide reductase activity, and thereby DNA synthesis in S-phase cells by depleting the nucleotide precursor pools of dCTP. No toxicity with this compound naturally present in the cell is observed contrary to hydroxyurea, 5 fluorouracil or aphidicolin which cause DNA damage.

**Figure 2:**
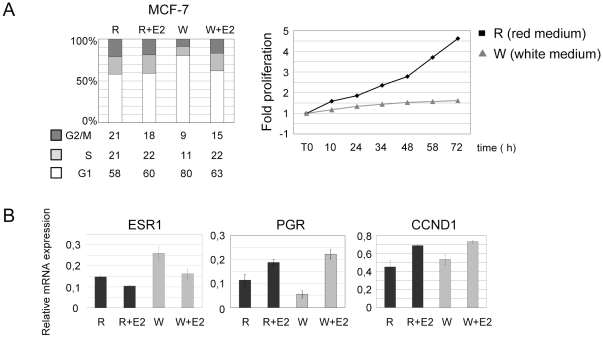
Steroid free medium induces an E2 reversible G1 block in ERα-positive MCF-7 cells. 1.5×10^6^ ERα-positive MCF-7 cells were cultivated in red medium (R) for 24 h, then medium was removed and cells were cultivated in red or white medium A) FACS analysis of MCF-7 cells after 4 days in red medium only (R), 3 days in red medium complemented with E2 10^−8^ M for 24 h, in white medium only for 4 days (W) or 3 days in white medium complemented with E2 10^−8^ M for 24 h (W+E2). For growth curves, 1×10^6^ ERα-positive MCF-7 cells were cultivated in red medium (R) for 24 h, then medium was removed and cells were cultivated in red or white medium. Cells were counted by trypan blue exclusion at different time points. B) *ESR1, PGR* and *CCND1* gene expression was analyzed by RT-qPCR.

**Figure 3:**
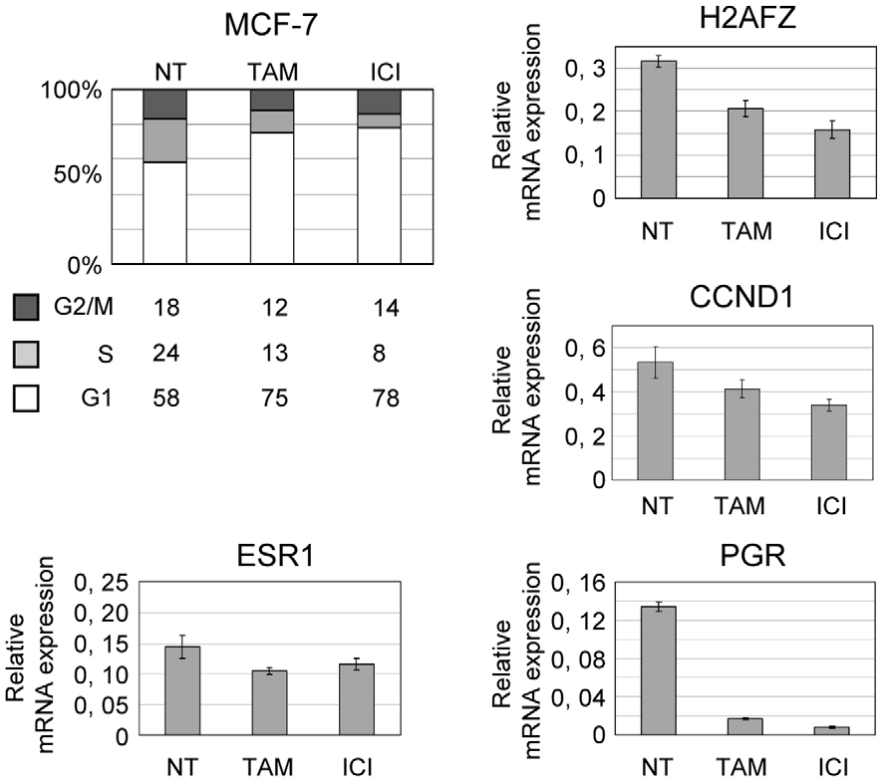
OH-TAM and ICI induced G1 cell cycle block and decreased ERα target gene expression. 2×10^6^ MCF-7 cells were cultivated in red medium for 24 h. OH-TAM or ICI were added to the medium at a final concentration of 1 µM. After 24 h cell cycle was analyzed by FACS and expression of ERα target genes by RT-qPCR.

Using quantitative RT- PCR we analyzed *ESR1*, *PGR*, *CCND1* and *H2AFZ* expression in synchronized cells. We found that relative mRNA levels of *ESR1* and *PGR* were about 2 fold higher in G1 than in S-phase (Figure 1B) and 2 to 3 fold higher in G1 than in G2/M phase. Control RPLO and GAPDH gene expression did not vary compared to G1 arrested cells. We note that variations in expression levels of *PGR* were similar to the ones reported by Nayaran *et al.* [16] in which serum starvation of T47D cells was employed. *CCND1* was also preferentially expressed in G1, although significant amounts of mRNA were detected in S and G2/M phases. Furthermore, *H2AFZ* expression in G1 corresponded to only ∼50% of *H2AFZ* mRNA levels measured in G2/M. We next analyzed gene expression in MCF-7 cells that had been synchronized by nocodazol before check-off and further growth (7 hours) to reach G1 (FACS, data not shown). Gene expression levels were identical to the ones recorded in the lovastatin G1 arrested sample (Figure 1C). We conclude that, in MCF-7 cells, transcription of the estrogen receptor gene and of ERα regulated target genes is cell cycle regulated.

### In steroid free medium ER**α** positive MCF-7 cells arrest in G1

Tumor derived MCF-7 cells are used to mimic hormone sensitive breast cancers. Two types of media are commonly used: red medium which is the standard medium for optimum growth (DMEM F12 and derivatives), and white medium which is used to analyze the expression of ERα target genes after addition of 17-ß estradiol (E2). White medium is phenol red depleted since phenol red is known to activate ERα gene regulation, and supplemented with steroid free (charcoal treated) serum. MCF-7 cells were cultivated in red and white medium with or without E2 reaching near 60 to 70% confluence (Figure 2A). Cell cycle profiles in red medium were almost identical with or without addition of E2 (10 nM). In contrast, after 3 days in white medium, a significant fraction of MCF-7 cells accumulated in G1 phase (80% of the cells, Figure 2A). A doubling time experiment (Figure 2A right panel) confirmed that these cells stopped growing. Thus MCF-7 cells cultivated in white medium are blocked in G1. This G1 block was reversed 24 h after addition of E2 (10 nM). Between 1 h and 24 h of E2 treatment we saw intermediate cell cycle profiles (data not shown). We next analyzed gene expression under different culture conditions (Figure 2B). E2 treatment in red medium reduced *ESR1* expression. *ESR1* is known to be down-regulated when ERα protein levels increase. This negative feedback loop lead to a rapid increase in *ESR1* transcription (15 min) and a subsequent decrease in mRNA levels as shown here 24 h after addition of E2. In white medium, cells were blocked in G1 and we observed an increase in *ESR1* expression compared to red medium culture conditions in agreement with our observation that *ESR1* expression was greatest in G1 (Figure 1B). Addition of E2 to either medium triggered a 50% reduction in *ESR1* mRNA levels. *PGR* and *CCND1* were activated by addition of E2 independently of the type of medium. After 24 h of E2 induction the expression levels of each gene tested were similar in red and in white media. Therefore, white medium culture conditions do not trigger any irreversible effect on cell cycle and gene expression. However, when analyzing the effects of drugs on gene expression, under steroid free culture conditions, cell cycle effects have to be taken into account.

### Regulation of gene expression by OH-Tam and ICI is cell cycle dependent

The principal strategy for inhibition of estrogen-dependent tumor growth is to block ERα signaling by anti-estrogen molecules (competitive hormone-therapy) such as Tamoxifen (OH-TAM) or Faslodex/ICI 182.780 (ICI). Numerous studies describe their molecular mechanisms of action in MCF-7 cell cultures. While it is clear that both classes of antiestrogens induce cells to arrest in G1, it has not been investigated whether their effects were cell cycle dependent [11], [19], [20], [21].

In agreement with previous reports [11], [20], OH-TAM and ICI 182.780 induced a strong G1 block in MCF-7 cells, and reduced expression levels of estrogen-regulated genes (Figure 3). By quantitative RT-PCR we observed a slight decrease of *ESR1* gene expression, whereas *PGR* expression was abolished and *CCND1* expression was reduced by 25% to 35%. Similarly, *H2AFZ* expression diminished by 30% and 40% after OH-TAM or ICI treatments, respectively.

**Figure 4:**
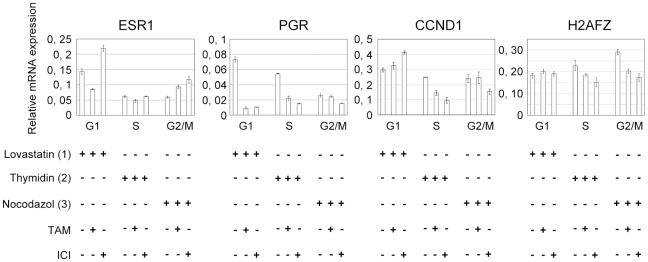
Effect of OH-TAM and ICI on gene expression is cell cycle dependent. 2×10^6^ cells were cultivated in red medium for 24 h. Cells were blocked in G1 by lovastatin 20 µM, 32 h (1), in S by thymidine 3 mM 24 h (2) and in G2/M by nocodazol 50 ng/ml, 24 h (3). Cell cycle arrested cells were treated by Tamoxifen (OH-TAM 1 µM, 24 h) or ICI 182.780 (ICI 1 µM, 24 h). (n = 2) one representative experiment is shown. Relative mRNA expression in G1, S or G2/M phases of ERα target with or without anti-estrogen treatment was analyzed by RT-qPCR.

Next, we investigated the impact of antiestrogens on gene expression in MCF-7 cells specifically blocked in G1 phase by lovastatin, in S phase by thymidine and G2/M phases by nocodazol. Arrested cells were treated with OH-TAM or ICI 1 µM for 24 h in the presence of cell cycle drugs and the effects on ERα regulated gene expression were analyzed in each cell cycle phase (Figure 4). We observed that OH-TAM and ICI both altered gene expression during the different cell cycle phases. Changes in transcription were not identical for these two antiestrogens. For example, while both drugs slightly stimulated *ESR1* transcription in G2/M, ICI activated *ESR1* and OH-TAM repressed *ESR1* expression in G1 arrested cells. The negative feedback induced by reduction in ERα protein levels in the presence of ICI thus stimulates *ESR1* transcription in G1. The inhibitory effect of both antiestrogens was most significant on *PGR* in G1 arrested cells. Interestingly, while neither OH-TAM nor ICI had an effect on *CCND1* expression in G1 or G2/M, OH-TAM reduced *CCND1* expression by more than 40% in S phase. In addition, OH-TAM and ICI treatments did not alter *H2AFZ* expression in G1 arrested cells but lead to a significant reduction of its expression in S and G2/M phases.

### Anti-estrogen action on cell proliferation is enhanced in cells blocked in S-phase

**Figure 5:**
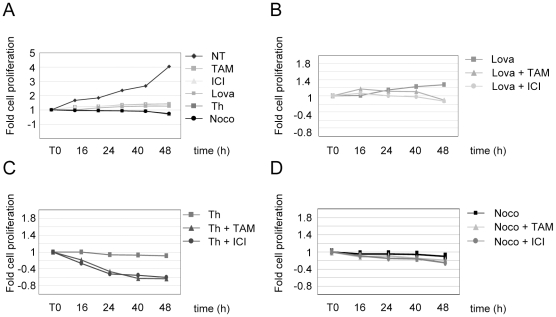
Effect of OH-TAM and ICI on cell proliferation is enhanced in S-phase. 3×10^5^ MCF-7 cells were split in 60 cm dishes and cultivated in red medium for 12 h. A) Cells were treated by lovastatin (20 µM, 32 h), thymidine (3 mM, 24 h) or nocodazol (50 ng/ml, 24 h) until T0. Cell proliferation was monitored for untreated (NT) or for OH-TAM (1 µM) or ICI (1 µM) treated cells. Cells were counted by trypan blue exclusion in triplicate at different time points. Number of cells at T0 was set to 1 and the doubling time was calculated. B-C-D). Cells were treated by lovastatin 20 µM for 32 h (B), by thymidine 3 mM, 24 h (C) or nocodazol 50 ng/ml, 24 h (D) until T0. Then, OH-TAM 1 µM or ICI 1 µM added to the medium and cells were counted by trypan blue exclusion in triplicate at different time points.

We examined the consequence of antiestrogen addition during each cell cycle phase on cell proliferation by a doubling time experiment (Figure 5). The doubling time of untreated MCF-7 cells (NT) is usually 28 h (Figure 2A and 5A). OH-TAM (TAM), ICI (ICI) (Figure 3) and lovastatin (Lova) (Figure 1) treatments induced a significant G1 block, but a portion of the cell population continued to proliferate (1.3 fold after 48 h) (Figure 5A). Thymidine (Th) and nocodazol (Noco) stop cell proliferation without any measurable impact on cell survival up to 48 h. Cells appear to arrest in their respective cell cycle phase (S and G2/M phases) respectively (Figure 1). No significant change in cell proliferation is observed when MCF-7 cells blocked in G1 by lovastatin (Figure 5B) or in G2/M (Figure 5D) by nocodazol were treated with OH-TAM or ICI during 48 h. However, both antiestrogens severely reduced the number of living cells when applied in S phase: after 24 h treatment only 50% of cells remained (Figure 5C).

**Figure 6:**
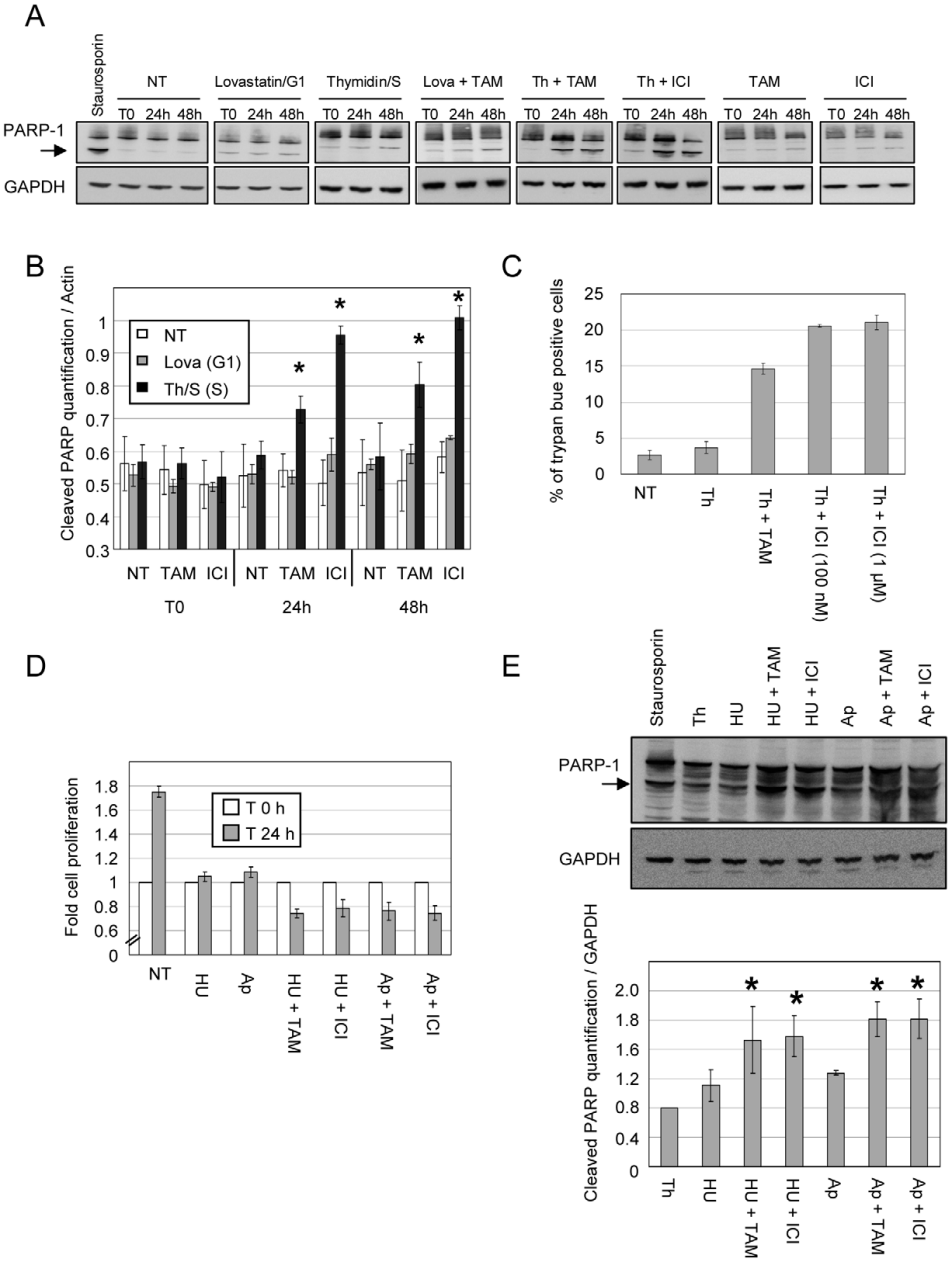
OH-TAM and ICI induced apoptosis specifically in S-phase in MCF-7 arrested cells. A) 3×10^5^ MCF-7 cells were split in 60 cm dishes and cultivated in red medium for 12 h. Cells were treated by lovastatin (20 µM, 32 h), thymidine (3 mM, 24 h) or nocodazol (50 ng/ml, 24 h) until T0. Accumulation of cleaved PARP (black arrow) was monitored by western blotting for untreated (NT) or for OH-TAM (1 µM) or ICI (1 µM) treated cells. B) Quantification analysis was performed using the ImageGauge 4.0 software. * increase >50% of cleaved PARP. C) 3×10^6^ cells were seeded in 10 cm dishes. After 12 h, cells were treated or not (NT) with 3 mM thymidine for 24 h. Cells were treated with thymidine (Th) or Tamoxifen 1 µM (Th + TAM), ICI 100 nM or 1 µM (Th + ICI) for 24 h. After trypsinisation, cells were counted by trypan blue staining. Total cell number was set to 100% (n = 2). D) 3×10^6^ cells were splited in 10 cm dishes. At T0 cells were treated with hydroxyl urea 1,5 mM or aphidicolin 1 µg/ml for 24 h. Then, cells were treated or not with Tamoxifen or ICI 1 µM for another 24 h. Cells were counted by trypan blue exclusion. Number of cells at T0 was set to 1 and the doubling time was calculated. E) Cells were treated as in (D). Accumulation of cleaved PARP (black arrow) was monitored by western blotting. Quantification analysis was performed using the ImageGauge 4.0 software. * increase >50% of cleaved PARP.

We thus determined whether cell cycle drugs alone or in combination with anti-estrogens induced apoptosis (Figure 6A). MCF-7 cells were treated as above, whole cell extracts were subjected to Western blotting using anti PARP-1 antibody. Cleaved PARP was quantified by Image gauge software and normalized by GAPDH expression (Figure 6B). 6 hours treatment with staurosporin at 1 µM was used as a positive control of apoptosis and quantification of cleaved PARP after this treatment was set to 1 [22]. Only cells treated with OH-TAM or ICI in S-phase (thymidine arrested cells) accumulated cleaved PARP to levels comparable to those detected in cells treated with staurosporin. Under all other conditions, anti-estrogen treatment alone or in G1 or G2/M arrested cells induced no or very little accumulation of cleaved PARP. In addition, trypan blue staining revealed that the percentage of trypan blue positive cells was >5 times higher in S-phase arrested cells treated with antiestrogens (∼20%) than in untreated or in thymidine arrested cells (Figure 6C).

Synchronizing MCF-7 cells with hydroxy urea (HU) 1,5 mM or aphidicolin (Ap) 1 µg/ml for 24 h prior to antiestrogen treatment also blocked cells in S-phase without any measurable impact on cell survival (Figure 6D). However, similarly to cells arrested by thymidine, addition of OH-TAM and ICI to HU or Ap treated cells reduced the number of living cells by inducing apoptosis (Figure 6D). The amount of cleaved PARP1 was about 50% greater in antiestrogen treated, S-phase arrested cells than in untreated cells arrested by HU or Ap (Figure 6E). Hence, the impact of antiestrogens on cell survival is potentiated in S-phase independently of the treatment used to synchronize cells. Furthermore, this reduction of cell survival correlated with a significant decrease in cyclin D1 transcription in cells treated with antiestrogens in S-phase (Figure 4A) and a clear reduction of the growth rate of these cells at 24 and 48 h (Figure 5). Taken together, these data clearly demonstrate that the impact of anti-estrogen treatment depends on the cell cycle phase.

## Discussion

We show that in MCF-7 cells the estrogen receptor and downstream target genes are cell cycle regulated with expression levels varying as much as three-fold between phases of the cell cycle. Thus, cell cycle effects have to be taken into account when interpreting data obtained from assays involving drugs that affect cell cycle progression. Indeed, under commonly employed culture conditions used to assess the effect of hormones on gene expression (steroid free or ‘white medium), MCF-7 cells arrest in G1. Importantly, several ERα target genes are overexpressed in G1 relative to G2/M. In particular, the apparent increase in *ESR1* gene expression may partially be a cell cycle effect. Interaction between unbound ERα and p21^WAF1^ has previously been proposed to have an antiproliferative effect [23]. This effect could be exacerbated by increased ERα levels in G1. Since cells return to cycle normally only after 24 h, such protein-protein interactions may modulate gene expression specifically in “white” medium.

We further demonstrate that antiestrogen effectiveness is largely influenced by the cell cycle. One of the most commonly used antiestrogens is Tamoxifen [24], also called SERM, for selective estrogen receptor modulator. Pure antagonists such as ICI 164.384 or ICI 182.780 have been developed to avoid undesirable side effects due to stimulating effects of SERMs in other tissues. We noticed significant variability in gene expression between OH-TAM and ICI treated cells in different cell cycle phases. For example, in G1, *ESR1* transcription was inhibited by OH-TAM but activated by ICI. Yet, in G2/M, OH-TAM and ICI both activated *ESR1* expression. It is likely that these differences stem from the different mode of action of these antiestrogens. OH-TAM bound ERα recognizes ERE sequences of target genes, but recruits several co-repressors in mammary tumor cells due to a conformational change induced by OH-TAM [25], [26]. ICI bound ERα is in a non-functional conformation and hydrolyzed by the proteasome – in any cell cycle phase.

We demonstrate for the first time that the effect of OH-TAM and ICI on target gene expression varies depending on cell cycle phase. One would expect OH-TAM and ICI to repress transcription, yet *CCND*1 transcription is not decreased when using OH-TAM or ICI in G1 cells. The repressive effect is only seen in S-phase. Inversely, these AEs do not affect *ESR1* transcription in S phase and differentially regulate *ESR1* in G1 or G2 phase.

Tamoxifen and ICI arrest cells in G0/G1 ([15], [27]; Figure 5). This anti-proliferative activity is associated with an inhibition of Cdk activity, a decrease in pRB phosphorylation, as well as a decrease in expression of several ERα target genes including Cyclin D1, c-Myc or Cyclin E [28], [29] but an increase in p21 and p27 expression [24], [30]. Cyclin D1, a key regulator of the G1/S transition and PI3K/AKT and MAPK signaling pathways, acts as mitogenic sensor in G1 [31], [32], [33]. *CCND1* is one of the most commonly overexpressed genes in breast cancer (up to 50% of breast cancers) [33], [34]. Its overexpression in mice leads to development of mammary carcinoma, while down-regulation induces resistance to cancer development [35], [36]. Its regulation is complex and upon E2 stimulation different transcriptional complexes can modulate *CCND1* transcription by direct ERα mediated genomic function but also as an indirect regulation of the activity of co-factors trough E2-induced non genomic effects [37].

Interestingly, antiestrogens repress transcription of all ERα target genes tested in S phase. This observation corroborates the more rapid and strong impact of antiestrogen treatments on cell proliferation in S-phase compared to similar treatments in G1 or in G2/M. Only in S-phase, MCF-7 cells induced massive apoptosis in the presence of 1 µM anti-estrogens. While it has been reported that OH-TAM can induce cell death at >5 µM in MCF-7 cells, little or no apoptosis was observed at 1 µM [38]. In vivo the percentage of apoptotic death within tumors was also low (about 5%) [39], [40]. Nevertheless, different signalling pathways have been implicated in OH-TAM or ICI-induced apoptosis (such as protein kinase C, c-MYC, p53 or MAP kinase) the exact mechanism is still unknown [41], [42]. Reduction in cyclin D1 transcription cannot explain this effect. It will be interesting to search for S-phase specific genes that are directly or indirectly ERα regulated and sensitive to antiestrogen treatments. Although not a direct target of ERα, the *PCNA* gene is a potential candidate [43]. Indeed, *PCNA* is upregulated in MCF-7 cells in the presence of E2 [7] and thus its regulation may be sensitive to antiestrogens. Possibly, replication may be defective due to reduced *PCNA* levels upon anti-estrogen treatment of cells in S-phase. Non functional *PCNA* has also been linked to apoptotic effects [44], [45]. The effect of antiestrogens in S-phase is most likely indirect by affecting numerous signalling pathways, including the AKT phosphorylation activity which activates Chk1 and other factors necessary for replication origin firing. Any perturbation of the frequency of replication origin firing will induce replicative stress which in turn activates p53 and downstream events, including apoptosis [46]. From a clinical point of view, the induction of apoptosis is an important component of breast cancer regression. The enhanced effects of antiestrogens specifically in S-phase, suggest that associating antiestrogens and cell cycle drugs represent a therapeutically attractive avenue.

## Materials and Methods

### Reagents

Estradiol, Thymidine, Nocodazol, Lovastatin (Mevinolin), Tamoxifen (OH-TAM), Hydroxy urea, Aphidicolin and Staurosporin were purchased from Sigma-Aldrich (Saint-Quentin Fallavier, France). ICI 182.780 (ICI) was purchased from TOCRIS (MO, USA).

### Cell lines and tissue culture

MCF-7 cells purchased from ATCC (passage No. 146, used up to 10 passages), were maintained in Dulbecco's modified Eagle's medium (DMEM) F-12 with Glutamax containing 50 mg/ml gentamicin, 1 mM sodium pyruvate and 10% heat-inactivated fetal calf serum (FCS) (Invitrogen). These media are called red (R) because of the presence of phenol red. For steroid free medium or white medium (W), cells were grown for 3 days in media without phenol red, 50 mg/ml gentamicin, 1 mM sodium pyruvate and 10% of serum stripped of endogenous steroids. Cells were treated or not with 10^−8^ M E2, 1 µM OH-TAM, 1 µM ICI, 20 µM Lovastatin, 3 mM Thymidine, 50 ng/ml Nocodazol, 1,5 mM Hydroxy urea, 1 µg/ml Aphidicolin for the indicated times. For ‘check-off’, MCF-7 cells were treated 24 h with 25 ng/ml of nocodazol. After mechanical detachment (check-off) cells were washed three times in PBS, one time with complete medium and seeded in a new dish.

### RNA extraction, reverse transcription and Quantitative PCR analysis

Total RNA was extracted using an RNeasy mini kit (Qiagen) and eluted with 35 µl of RNAase-free water. First strand cDNA was generated using 2 µg of total RNA in a reaction containing random oligonucleotides as primers with the ThermoScript RT-PCR system (Invitrogen). Real-time PCR was performed on Mastercycler® ep *realplex* ^4^ (Eppendorf) using the platinium SYBR Green qPCR SuperMix (Invitrogen) according to the manufacturer's instructions. Amplification conditions: 1 min at 50°C, 3 min at 95°C followed by 40 cycles (20 s at 95°C, 20 s at 60°C, 20 s at 72°C). The following primer pairs were used to amplify cDNAs after reverse transcription experiment ESR1: 5′- TGGAGATCTTCGACATGCTG - 3′ and 5′- TCCAGAGACTTCAGGGTGCT-3′, PGR: 5′-CTTAATCAACTAGGCGAGAG-3′ and 5′-AAGCTCATCCAAGAATACTG-3′ H2AFZ: 5′-CCTTTTCTCTGCCTTGCTTG-3′ and 5′-CGGTGAGGTACTCCAGGATG-3′, CCND1: 5′- GCGTCCATGCGGAAGATC-3′ and 5′-ATGGCCAGCGGGAAGAC-3′. Expression of PRPLP0 was used as control. PRPLP0: 5′-TGGCAGCATCTACAACCCTGAA-3′ and 5′- ACACTGGCAACATTGCGGACA- 3′. Experiments were repeated two times.

### Western blotting

Samples were separated by SDS–polyacrylamide gel electrophoresis. Anti-PARP-1 antibody was purchased by Alexis Biochemicals (ALX 210-895) and was used at 1/2000 dilution, anti-GAPDH from Millipore (MAB 374) was used at 1/1000 dilution. Quantification analysis was performed using the ImageGauge 4.0 software.

### Flow cytometry (FACS)

Cells were harvested directly from culture plates. After centrifugation at 1400rpm, 4°C, 5 mn, the pellet was washed with PBS/BSA 1% and resuspended in 500 ml PBS. Cells were fixed by adding 1.5 ml 100% cold ethanol and left at least 2 h at −20°C. Then, cells were washed with 4 ml PBS/BSA 1%. After centrifugation (4 min, 1400 rpm 4°C, 5 mn) the pellet was resuspended in FACS buffer: 500 µl PBS, RNAse A 1 mg/ml, Propidium Iodide 10 µg/ml and incubated 30 min, 37°C in the dark. Cell cycle profile was analysed with a Facscalibur apparatus (BD Biosciences).
